# NiFeMn-Layered Double Hydroxides Linked by Graphene as High-Performance Electrocatalysts for Oxygen Evolution Reaction

**DOI:** 10.3390/nano12132200

**Published:** 2022-06-27

**Authors:** Ze Wang, Qianyu Zhou, Yanni Zhu, Yangfan Du, Weichun Yang, Yuanfu Chen, Yong Li, Shifeng Wang

**Affiliations:** 1 Innovation Laboratory of Materials for Energy and Environment Technologies, Institute of Oxygen Supply, Tibet University, Lhasa 850000, China; wangze@utibet.edu.cn (Z.W.); zhouqianyu@utibet.edu.cn (Q.Z.); zhuyanni1210@163.com (Y.Z.); duyangfan@utibet.edu.cn (Y.D.); yangwec98@163.com (W.Y.); 2 Key Laboratory of Cosmic Rays, Tibet University, Ministry of Education, Lhasa 850000, China; 3 State Key Laboratory of Electronic Thin Films and Integrated Devices, University of Electronic Science and Technology of China, Chengdu 610054, China

**Keywords:** NiFeMn, layered double hydroxides, oxygen/hydrogen evolution reaction, electrocatalysis, water splitting

## Abstract

Currently, precious metal group materials are known as the efficient and widely used oxygen evolution reaction (OER) and hydrogen evolution reaction (HER) catalysts. The exorbitant prices and scarcity of the precious metals have stimulated scale exploration of alternative non-precious metal catalysts with low-cost and high performance. Layered double hydroxides (LDHs) are a promising precursor to prepare cost-effective and high-performance catalysts because they possess abundant micropores and nitrogen self-doping after pyrolysis, which can accelerate the electron transfer and serve as active sites for efficient OER. Herein, we developed a new highly active NiFeMn-layered double hydroxide (NFM LDH) based electrocatalyst for OER. Through building NFM hydroxide/oxyhydroxide heterojunction and incorporation of conductive graphene, the prepared NFM LDH-based electrocatalyst delivers a low overpotential of 338 mV at current density of 10 mA cm^−2^ with a small Tafel slope of 67 mV dec^−1^, which are superior to those of commercial RuO_2_ catalyst for OER. The LDH/OOH heterojunction involves strong interfacial coupling, which modulates the local electronic environment and boosts the kinetics of charge transfer. In addition, the high valence Fe^3+^ and Mn^3+^ species formed after NaOH treatment provide more active sites and promote the Ni^2+^ to higher oxidation states during the O_2_ evolution. Moreover, graphene contributes a lot to the reduction of charge transfer resistance. The combining effects have greatly enhanced the catalytic ability for OER, demonstrating that the synthesized NFM LDH/OOH heterojunction with graphene linkage can be practically applied as a high-performance electrocatalyst for oxygen production via water splitting.

## 1. Introduction

Since the third technological revolution, energy supply has acquired special importance in national economies. With the rapid growth of industrial civilization, the problems of energy shortages and environmental pollution are becoming more and more prominent. Thus, the development of new energy sources becomes the most important thing in social developments [1,2,3]. The oxygen evolution reaction (OER) is critical for the advancement of different electrochemical applications such as energy storage and water splitting [4,5,6]. However, the kinetics of the OER reaction is severely hampered by multiple electron transfer. Thus, it is of great significance to develop high-performance OER catalysts.

Recently, great efforts have been made for developing high-performance OER electrocatalysts, which can be classified as follows: (1) the most common OER catalysts are prepared with noble metals, such as Ru-based and Ir-based catalysts. It showed outstanding electrochemical performance, but the high cost, scarcity, and easy poisoning limit their extensive application [7,8,9,10,11,12]. (2) Based on related works, non-precious metals (Fe, Cu, Co, Ni, Mn, etc.) carbon hybrid materials have emerged as a promising alternative to Pt-based ORR catalysts. They show great OER catalytic properties, which is close to commercial noble metal catalysts [13,14,15,16,17,18,19]. Given the above situation, developing catalysts that are more active, stable, and cost-effective than the commercial RuO_2_ is desirable, but still challenging.

Layered double hydroxides (LDHs) are a type of metal hydroxides composed of two or more metal elements [20,21,22,23]. It is an ideal precursor to prepare OER catalyst due to the following advantages: (1) LDHs are rich in raw materials, low prices, and simple production process; (2) its abundant metal ions can be turned into efficient catalytic active sites to accelerate the OER rate. To achieve this goal, the most common metal components used for the LDH based catalysts should be inexpensive and involve earth-abundant elements [24,25,26,27].

In this work, a low-cost NiFeMn LDH template strategy is proposed for fabricating high-performance OER catalysts. The LDHs consisting of Ni, Fe, Mn elements exhibit a layered structure with micrometer-sized sheets and nanometer-sized rods, which are beneficial to the surface-sensitive reactions. When the NFM LDH was treated by NaOH, NFM LHD/OOH heterojunctions were built with strongly coupled interfaces, which is thought to reform the local electronic environment and accelerate the inter-band charge transfer. XPS analysis revealed that the amount of the highly oxidative Fe^3+^ and Mn^3+^ substantially grew in the heterojunction, which would offer an increased number of active sites for enhanced catalytic activities. However, the prepared NFM LDHs showed high charge transfer resistance, which is reflected from the large arc in the electrochemical impedance spectroscopy (EIS) test, impeding the practical application. To address this issue, graphene is incorporated into NFM LDH-based electrocatalysts, substantially reducing the resistance and boosting the electrocatalytic performance. Finally, NFM LDH/OOH heterostructure with graphene linkage exhibits a low overpotential of 338 mV at a current density of 10 mA cm^−2^ with a small Tafel slope of 67 mV dec^−1^, which is superior to those of commercial RuO_2_ catalyst for OER. The high performance arising from the combining effects has demonstrated that the synthesized NFM LDH-based electrocatalyst can be practically used as a high-performance OER electrocatalyst for water splitting. The strategies of building heterostructures and linking the functional materials with conductive 2D materials pave the way for exploring high efficiency and low cost electrocatalysts for hydrogen and oxygen generation through water-splitting.

## 2. Materials and Methods

### 2.1. Synthesis of NFM-Layered Double Hydroxides and Their Derivatives

#### 2.1.1. Materials

Nickel chloride (NiCl_2_·6H_2_O) was purchased from West Asia Chemical Technology Company (Linyi, Shandong Province, China), while Ferrous chloride (FeCl_2_·4H_2_O) was purchased from Tianjin Guangfu Fine Chemical Company (Tianjin, China), and Manganese chloride (MnCl_2_·4H_2_O) and Sodium hydroxide (NaOH) were bought from Fuchen Chemical Reagent Co., Ltd. (Tianjin, China). Urea was obtained from Tianjin Regent Chemicals Co., Ltd. (Tianjin, China), and graphene oxide was obtained from Nanjing XFNANO Materials Tech Co., Ltd. (Nanjing, Jiangsu Province, China). All chemicals were used directly in the process and deionized water was used in the experiments.

#### 2.1.2. Preparation of NiFeMn-Layered Double Hydroxides

NiFeMn-layered double hydroxide (denoted as NFM LDH) was prepared by the following method. Typically, 0.596 g ferrous chloride, 0.894 g manganese chloride, 1.192 g nickel chloride and 1.788 g of urea were added to 40 mL deionized water. Then, the above solution was mixed and transferred into a hydrothermal kettle (120 °C for 12 h). Finally, the NFM LDH was obtained by centrifugation (5000 rpm) and dried at 60 °C for 12 h.

#### 2.1.3. Preparation of NFM LDH with Graphene

NiFeMn LDH with graphene connection (denoted as NFM LDH-G) was obtained by the following method. Typically, 30 mg graphene oxide, 0.596 g ferrous chloride, 0.894 g manganese chloride, 1.192 g nickel chloride and 1.788 g of urea were added to 40 mL deionized water. Then, the above solution was mixed and transferred into a hydrothermal kettle (120 °C for 12 h). Finally, the NFM-G LDH was obtained by centrifugation at 5000 rpm and dried at 60 °C for 12 h.

#### 2.1.4. Preparation of NFM LDH/OOH Heterostructures

NFM LDH/OOH heterostructure (denoted as NFM LDH/OOH) was fabricated by the following method. A total of 25 mg NFM LDH was added into 40 mL sodium hydroxide solution (1 M). Then, the above solution was stirred at 700 rpm for 24 h. Finally, the NFM LDH/OOH was obtained by centrifugation at 5000 rpm and dried at 60 °C for 12 h.

#### 2.1.5. Preparation of NFM LDH/OOH-G

NFM LDH/OOH heterostructure with graphene linkage (denoted as NFM LDH/OOH-G) was obtained by the following method. A total of 25 mg NFM LDH-G was added into 40 mL sodium hydroxide solution (1 M). Then, the above solution was stirred at 700 rpm for 24 h. Finally, the NFM LDH/OOH-G was obtained by centrifugation at 5000 rpm and dried at 60 °C for 12 h.

**Scheme 1:**
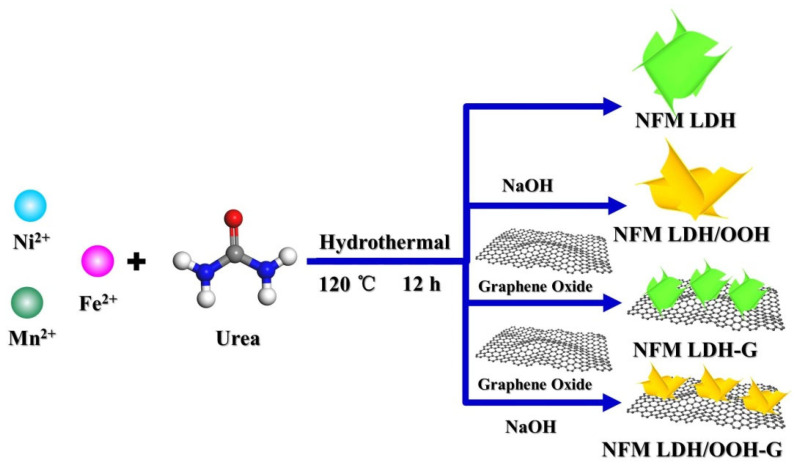
Preparation process of NFM LDH, NFM LDH/OOH, NFM LDH-G, NFM LDH/OOH-G.

The preparation of NFM LDH and its derivatives is summarized in Scheme 1. Firstly, Fe^2+^, Ni^2+^, Mn^2+^, and urea were added to deionized water. Then, the above solution was heated to 120 °C for 12 h in a hydrothermal kettle.

### 2.2. Characterizations and Testing

#### 2.2.1. Characterizations

The structural property was examined by X-ray diffraction (XRD) performed on a D/max 2500 X-ray powder diffractometer ( Rigaku Corporation, Tokyo, Japan), using a Co Kα radiation source with a wavelength of 1.79026 Å (at 40.0 kV and 40.0 mA) over the 2θ range from 10° to 90° at the scan rate of 5°/min. Scanning electron microscopy (SEM) was employed to probe the microstructure as well as the morphology of the samples, using a Hitachi SU8020 microscope (Hitachi Ltd., Tokyo, Janpan). High-resolution transmission electron microscopy (HRTEM) and elemental mapping was performed on a JEM-F200X field emission transmission electron microscope (JEOL Ltd., Tokyo, Japan). The X-ray photoelectron spectroscopy (XPS) spectra were recorded by an ESCALAB 250XI (Thermo Fisher Scientific Inc., Waltham, MA, USA) using a 200 W monochromator with Al Kα radiation as the excitation source, and the binding energies were calibrated by C 1s at 284.8 eV.

#### 2.2.2. Electrocatalytic Performance Testing

Electrochemical measurements were performed on a CHI-650E electrochemical workstation (Shanghai CH Instruments Co., Ltd., Shanghai, China). A three-electrode system was used in the experiment with a glassy carbon electrode (GCE) as the working electrode, a saturated calomel electrode (saturated KCl) as reference electrode, and a graphite rod as counter-electrode. The electrocatalytic properties of as-prepared OER catalysts (NFM LDH, NFM LDH/OOH, NFM LDH-G, NFM LDH/OOH-G, and RuO_2_) were tested by linear sweep voltammetry (LSV) and electrochemical impedance spectroscopy (EIS). The electrochemical test was preceded by an activity sweep using cyclic voltammetry at 0.1 V/s for 20 revolutions. In the LSV test, the scan potential window and scan rate were set at 0 to 0.8 V and 5 mV/s, respectively. EIS measurements were conducted from 1.0 × 10^−3^ to 1.0 × 10^5^ HZ. The electrolyte was 1 M KOH.

## 3. Results

**Figure 1:**
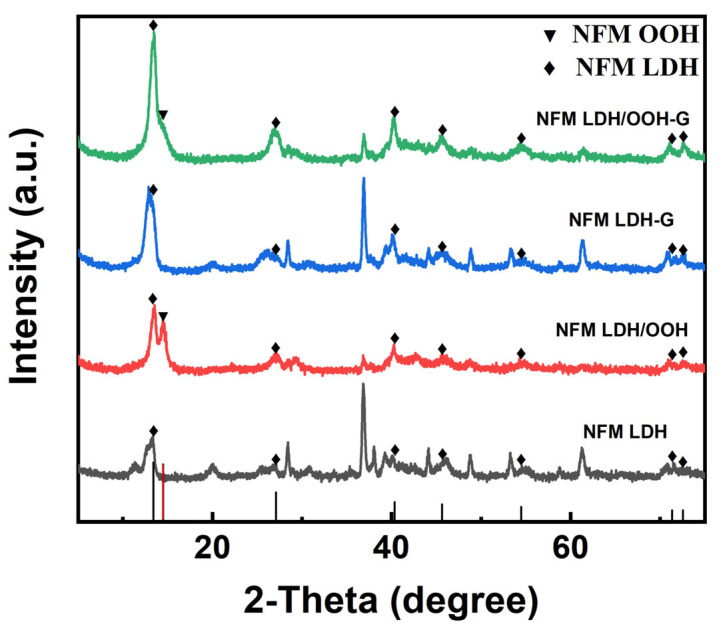
XRD patterns of NFM-LDH and its derivatives.

The structural properties of the as-prepared NFM-LDH and its derivatives were examined by XRD, as shown in Figure 1. The diffraction peaks located at 2θ = 13.4°, 27.1°, 40.4°, 45.6°, 54.5°, 71.3°, and 72.5° are well indexed to the (003), (006), (012), (015), (018), (0015), and (113) diffraction planes of NFM hydroxide (JCPDS: #51-0463) for all samples, respectively. In other words, NFM-LDHs have been successfully prepared. However, for the sample that was treated with NaOH, an obvious peak at 2θ = 14.5° emerges, indicated by the inverted triangles in Figure 1, which is aligned to JCPDS: #29-0889 and demonstrates the formation of NFM oxyhydroxides on the NFM LDHs.

**Figure 2:**
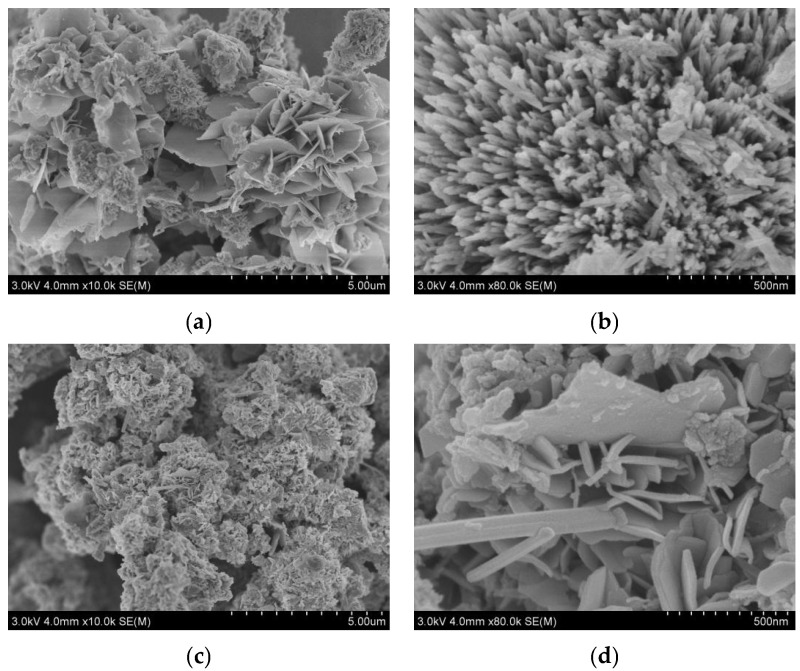

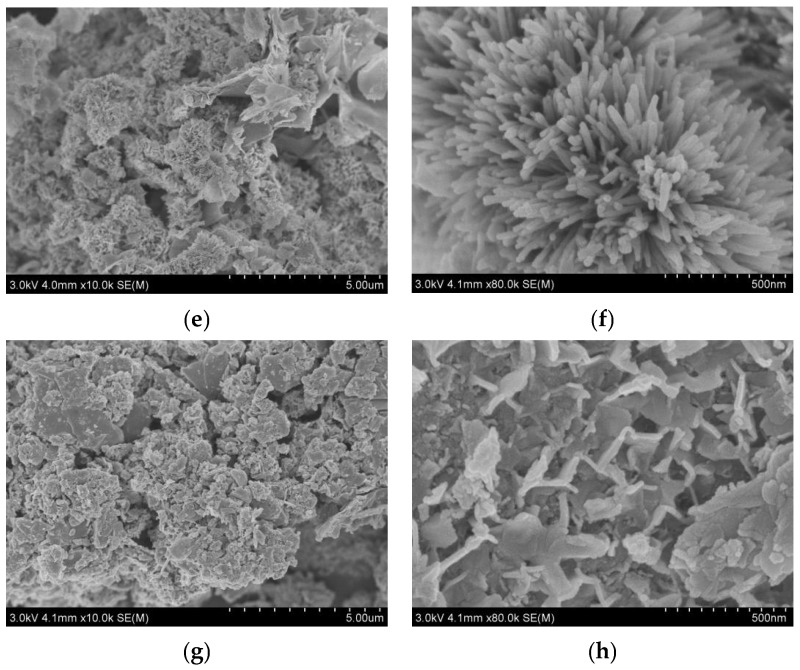
SEM images of (**a**,**b**) NFM LDH, (**c**,**d**) NFM LDH/OOH, (**e**,**f**) NFM LDH-G, (**g**,**h**) NFM LDH/OOH-G.

The morphologies of NFM LDH and its derivatives were characterized by scanning electron microscope and high-resolution transmission electron microscopy analysis. As shown in Figure 2a,b,e,f the NFM LDH and its compound with graphene exhibit almost the same morphology consisting of micrometer-sized sheets and nanometer-sized rods. These micro- and nano-structures present large surface area, which is beneficial to the surface-sensitive reactions. When the samples were treated with NaOH, the morphology did not change a lot, as shown in Figure 2c,d,g,h.

**Figure 3:**
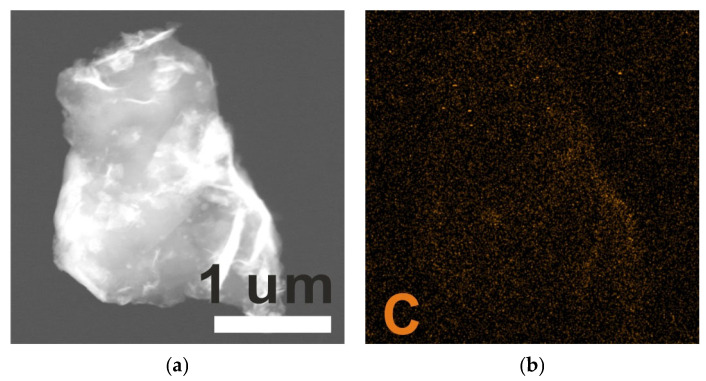

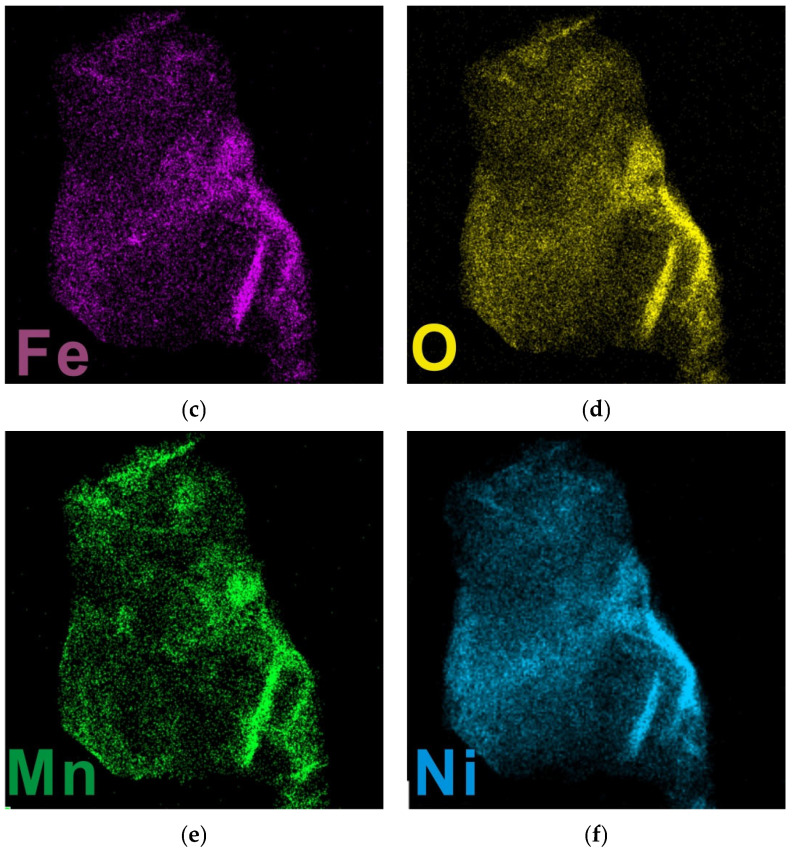
Elemental mapping of (**a**) NFM LDH/OOH-G and the distribution of (**b**) C, (**c**) Fe, (**d**) O, (**e**) Mn, and (**f**) Ni.

Furthermore, elemental mapping by energy dispersive spectroscopy (EDS) was used to study the spatial distribution of each element. As shown in Figure 3, Ni, Fe, Mn, C, and O elements are highly dispersed and well distributed in the NFM LDH/OOH-G sample, confirming the successful fabrication of NFM hydroxides and oxyhydroxides hybrids and the addition of graphene.

**Figure 4:**
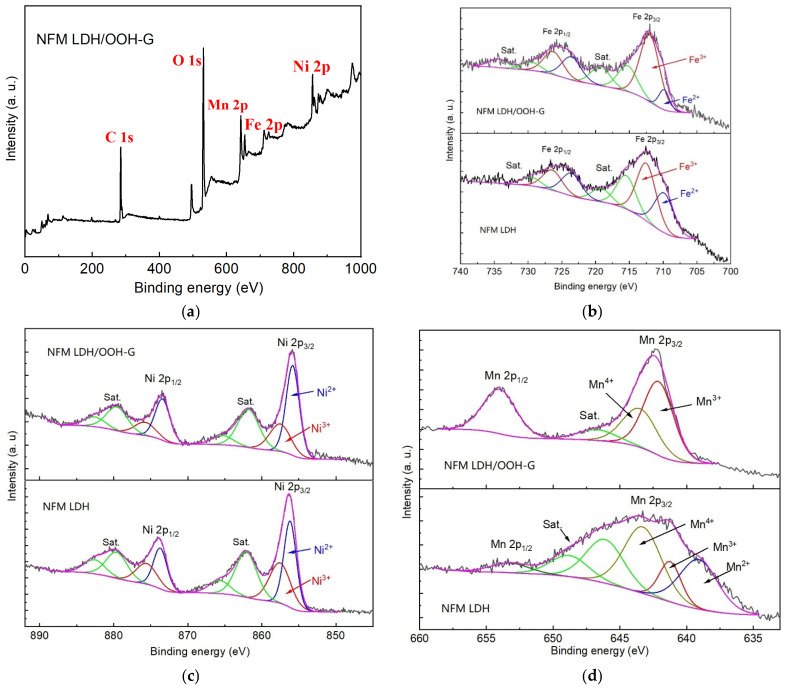
X-ray photoelectron spectrum of (**a**) NFM LDH/OOH-G and high-resolution spectra of (**b**) Fe, (**c**) Ni (C), (**d**) Mn for NFM LDH (lower) and NFM LDH/OOH-G (upper), respectively.

XPS measurements were carried out to identify and quantify the chemical composition and oxidation states of the elements present in the prepared electrocatalysts. The full survey XPS spectrum, as shown in Figure 4a, confirms the co-existence of Ni, Fe, Mn, O, and C elements in the samples, which is in good agreement with the EDS results. As displayed in Figure 4b, the characteristic peak of Fe 2p_3/2_ is located at about 711 eV, and that of 2p_1/2_ is situated at around 725 eV, while the respective shake-up satellites (marked as Sat.) are located approximately 8 eV higher than the corresponding main peaks. By performing peak-fitting deconvolutions, the measured binding energies of Fe 2p_3__/2_ can be divided into two peaks at 709.76–709.87 eV and 711.63–712.48 eV, which are assigned to Fe^2+^ and Fe^3+^, respectively [28,29]. Additionally, by comparing the upper curve (NFM LDH/OOH-G) and the lower (NFM LDH) of Figure 4b, the Fe^2+^/Fe^3+^ ratio is calculated to be 0.16 and 0.69, respectively, implying an increase in the Fe^3+^ species after NaOH treatment and thus suggesting the formation of FeOOH. Researchers found previously that the OER activity trend of 3d transition metal divalent cations was sorted by Mn^2+^ < Fe^2+^ < Co^2+^ < Ni^2+^ [30]. Later, several studies revealed that Fe^3+^ could enhance significantly the OER activities for Ni-based catalysts in the following ways: (i) improving the electrical conductivity; (ii) shortening the bond length of oxygen bridge; (iii) promoting Ni^2+^ to an even high oxidation state during the O_2_ evolution [31]. Figure 4c shows the high-resolution XPS spectra of Ni 2p. It is clearly observed that the peaks at 855.83 eV and 857.51 eV are ascribed to the 2p_3/2_ of Ni^2+^ and Ni^3+^, respectively, and their satellite peaks locate at 861.80 eV and 865.31 eV. For Ni 2p_1/2_, the peaks occur at 873.40 eV and 875.76 and are attributed to the Ni^2+^ and Ni^3+^ species with their satellite peaks at 879.57 eV and 882.59 eV, respectively [32,33,34]. The lower curve of Figure 4d displays the XPS spectrum of Mn 2p, which can be separated into Mn^2+^, Mn^3+^, and Mn^4+^ components at 639.09, 641.23, and 643.28 eV, respectively [35,36]. However, while the samples were treated with NaOH, as shown in the upper curve of Figure 4d, Mn^2+^ species disappeared and Mn^3+^ valence states substantially increased with a growing ratio of Mn^3+^ to total oxidation states from 16.7% to 62.5%, indicating the transformation from Mn LDH to MnOOH. The rich Mn^3+^ and Mn^4+^ oxidation states can provide more active sites [37], promoting the OER process. In conclusion, the prepared NFM LDH/OOH via alkali treatment generates a considerable number of high-valence Fe^3+^ and Mn^3+^ species, which enable NFM LDH to possess more active sites and become more active, favoring the OER activities.

**Figure 5:**
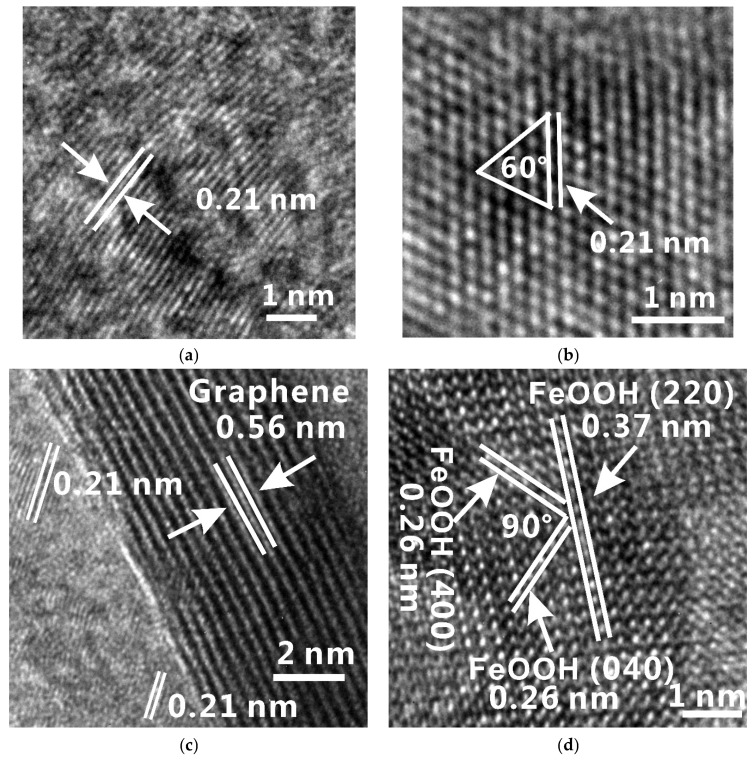

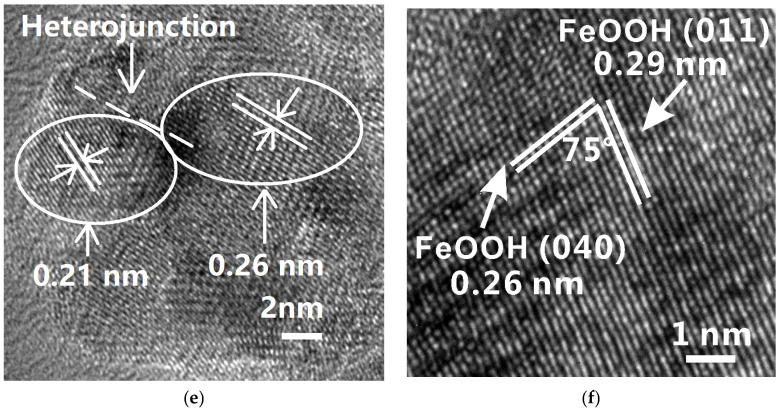
HRTEM images of (**a**) NFM LDH, (**b**,**c**) NFM LDH-G, (**d**,**e**) NFM LDH/OOH, (**f**) NFM LDH/OOH-G.

Figure 5 illustrates the HRTEM images of NFM LDH and its derivatives, respectively. The lattice spacing of 0.21 nm in Figure 5a,b is well associated with the (015) plane of Ni_5_._6_Fe_2_._36_(OH)_16_ in NFM LDHs, and the angle of 60° between the fringes in Figure 5b demonstrates the hexagonal structure of the as-prepared NFM LDHs. The spacing of 0.56 nm in Figure 5c,e originates from the contraction of graphene oxide (GO) while it is reduced (rGO) [38]. It is also observed that the rGO and NFM LDH are linked together, which is beneficial for OER electrocatalysis, since rGO possesses good conductivity [39]. After NaOH treatment, the angle between the fringes changes from 60° to 90°, implying a phase transition, as shown in Figure 5d. Moreover, the characteristic spacings of 0.26 nm, 0.26 nm, and 0.37 nm indicated by the white lines in Figure 5d are well indexed to the (400), (040), and (220) lattice planes of FeOOH crystals, respectively [40]. Additionally, Figure 5e shows the formation of NFM LDH/OOH heterojunction after NaOH treatment, mirrored by the characteristic spacings of 0.21 nm and 0.26 nm, which are assigned to the NFM LDH (015) plane and the FeOOH (400) plane, respectively. As shown in Figure 5e, the two crystal domains (indicated by the circles) originating respectively from the NFM LDH and oxyhydroxide contact each other, and the lattice atoms are connected, between which the heterojunction of NFM LDH/OOH is built (indicated by the dashed line). The fringe spacings of 0.29 nm and 0.26 nm with a dihedral angle of 75° in Figure 5f also originate from the (011) and (040) planes of FeOOH in NFM LDH/OOH-G, respectively. It was reported that the presence of FeOOH with highly oxidative Fe^(3 + δ)+^ species could reform the local electronic environment of NFM LDH through strong interfacial interactions, enabling the enhanced kinetics of inter-band charge transfer and generating the synergy catalytic effect between the two [33], thus promoting the OER electrocatalysis.

**Figure 6:**
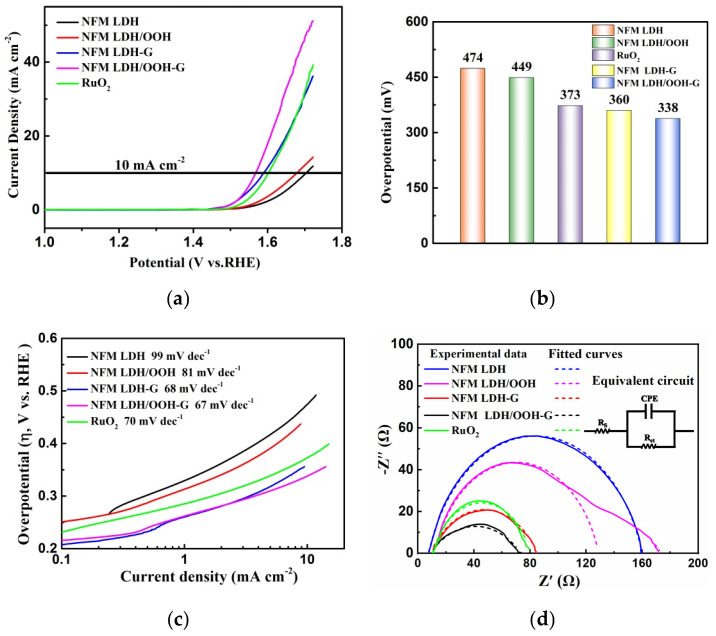
Electrocatalytic performance tests: (**a**) LSV plot; (**b**) Overpotential; (**c**) Tafel slope diagram; (**d**) EIS spectrum (solid lines represent the experimental data and the dashed lines denote the fitted curves).

**Figure 7:**
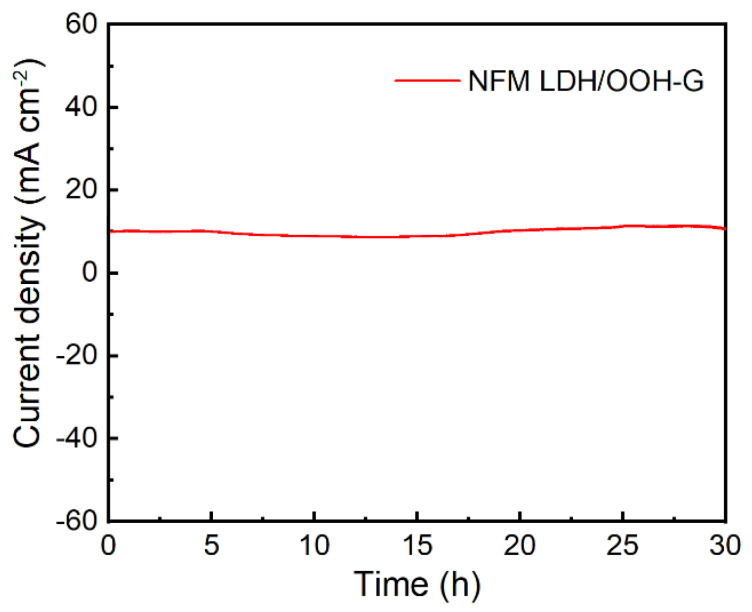
Chronopotentiometric curve of NFM LDH/OOH-G at the current density of 10 mA cm^−2^ in 1 M KOH solution.

The oxygen evolution activity of NFM LDH and its derivatives were evaluated by linear sweep voltammetry (LSV) and electrochemical impedance spectroscopy (EIS) in 1 M KOH solution. As shown in Figure 6a,b, NFM LDH/OOH heterojunction delivers an overpotential of 449 mV at 10 mA cm^−2^, which is 25 mV lower than that of pure NFM LDH, manifesting an enhanced OER electrocatalytic activity. The incorporation of graphene into the NFM LDH and its oxyhydroxide heterojunction further lowers the overpotential to, respectively, 360 mV and 338 mV, which are both smaller than that of commercially used RuO_2_ catalyst (373 mV), suggesting a substantial improvement in electrocatalytic performance. The comparison of the overpotential for the prepared NFM LDH-based electrocatalysts, as well as the commercial RuO_2_, is summarized in Figure 6b. To evaluate the OER kinetics of the catalysts, the Tafel slope was calculated from the LSV curve with the Tafel equation (η=a+blogj,  where *η* is the overpotential, *j* is the current density, and *b* is the Tafel slope). As shown in Figure 6c, NFM LDH/OOH heterojunction with graphene connection exhibits the smallest Tafel slope of 67 mV dec^−1^, smaller than those of NFM LDH/graphene (68 mV dec^−1^), RuO_2_ (70 mV dec^−1^), NFM LDH/OOH heterostructure (81 mV dec^−1^), and NFM LDH (99 mV dec^−1^), indicating the superior charge transfer coefficient of NFM LDH/OOH heterojunction and the graphene linkage. EIS was further carried out to deeply understand the OER performance of the prepared electrocatalysts. In general, the diameter of the semicircle in the corresponding EIS diagram represents the charge transfer resistance (*R_ct_*), reflecting the catalytic dynamic performance. A smaller *R_ct_* means faster electron transfer at the interface between the electrolyte and electrocatalyst. As shown in Figure 6d, it is obviously seen that the NFM LDH/OOH heterojunction shows a smaller arc (117 Ω) than NFM LDH (152 Ω), suggesting a faster charge transfer, which is consistent with the findings of enhanced kinetics of charge transfer due to the heterojunction [33]. Moreover, the incorporation of conductive graphene substantially reduces the resistance, reflected by the significant reduction of the semicircles from around 152 Ω to 72 Ω (53.9% reduction) for NFM LDH-G, and from 117 Ω to 66 Ω (43.6% reduction) for NFM LDH/OOH-G, which endows the NFM LDH and NFM LDH/OOH heterostructure excellent electronic transport behavior. In contrast, the charge transfer resistance of commercial RuO_2_ is measured as 70 Ω, a little bit larger than that of NFM LDH/OOH-G. The much conductive graphene contributes to the improvement of electrical conductivity of the NFM LDH-related catalysts, and promotes the OER electrocatalytic activities of the as-prepared layered catalysts. The result is in accordance with the corresponding low overpotential and small Tafel slope. In addition, enduring stability is another important factor in evaluating the performance of catalyst materials. As shown in Figure 7, the prepared NFM LDH/OOH-G could run steadily at the current density of 10 mA cm^−2^ for 30 h, thus demonstrating its superior stability.

Table 1 lists the comparison of our work with other reported non-noble OER catalysts. It is seen that the prepared NFM LDH/OOH-G electrocatalyst is comparable to the most efficient non-noble catalysts for OER ever reported, showing great potential in practical application.

## 4. Conclusions

In this study, layered double hydroxides consisting of Ni, Fe, and Mn elements (NFM LDHs) were fabricated via the hydrothermal method, which were used as OER electrocatalysts for water-splitting for hydrogen and oxygen production. To boost the OER efficiency, strategies of NFM LDH/OOH heterostructure construction and graphene incorporation were proposed. Building NFM LDH/OOH heterostructures with strong interfacial interactions is thought to enable the modulated electronic property and endow the accelerated charge transfer. In addition, the number of high-valence Fe^3+^ and Mn^3+^ species after alkali treatment is greatly increased, which could provide more active sites and become more active during the OER process. Adding conductive graphene lowers significantly the charge transfer resistance, accelerating the electron transfer velocity at the interface between the electrolyte and electrocatalyst. The synergetic effects endow the prepared NFM LDH/OOH with graphene linkage a substantially low overpotential of 338 mV with a small Tafel slope of 67 mV dec^−1^ for OER, which is better than those of commercially used RuO_2_ catalyst. The results have demonstrated that the synthesized NFM LDH-based electrocatalyst through heterostructure construction and conductive 2D materials incorporation can be applied as a high-performance OER electrocatalyst for water splitting for hydrogen and oxygen generation.

## Figures and Tables

**Table 1:** Comparison of the characteristic OER performance between the prepared NFM LDH/OOH-G and other reported non-noble electrocatalysts in the literature.

Electrocatalysts	Overpotential at 10 mA cm^−2^	Tafel Slope (mV dec^−1^)	Reference
NGNF	340 mV	93.2	[41]
Co_2_P NP	364 mV	78	[42]
LSCFM	343 mV	63	[43]
NiCo_2_O_4_/Ti	353 mV	61	[44]
VB12Mo@NC	396 mV	79	[45]
NFM LDH/OOH-G	338 mV	67	This work

## Data Availability

Not applicable.
